# Association between metabolic syndrome and myocardial infarction among patients with excess body weight: a systematic review and meta-analysis

**DOI:** 10.1186/s12889-024-17707-7

**Published:** 2024-02-12

**Authors:** Zahra Sedaghat, Soheila Khodakarim, Seyed Aria Nejadghaderi, Siamak Sabour

**Affiliations:** 1 Student Research Center, Department of Epidemiology, School of Public Health and Safety, Shahid Beheshti University of Medical Sciences, Tehran, Iran; 2 Department of Biostatistics, School of Medicine, Shiraz University of Medical Sciences, Shiraz, Iran; 3 School of Medicine, Shahid Beheshti University of Medical Sciences, Tehran, Iran; 4 Systematic Review and Meta-analysis Expert Group (SRMEG), Universal Scientific Education and Research Network (USERN), Tehran, Iran; 5 Department of Clinical Epidemiology, School of Public Health and Safety, Shahid Beheshti University of Medical Sciences, Tehran, Iran

**Keywords:** Metabolic syndrome, Myocardial infarction, Obesity, Overweight, Systematic review, Meta-analysis

## Abstract

**Background:**

Cardiovascular diseases (CVDs) are a major cause of morbidity and mortality worldwide. Controversial views exist over the effects of metabolically unhealthy obesity phenotypes on CVDs. This study aimed to perform a meta-analysis to assess the association between metabolic syndrome and myocardial infarction (MI) among individuals with excess body weight (EBW).

**Methods:**

We searched PubMed/Medline, Scopus, and Web of Science databases as of December 9, 2023. Cohort studies involving patients with overweight or obesity that reported the relevant effect measures for the association between metabolic syndrome and MI were included. We excluded studies with incomplete or unavailable original data, reanalysis of previously published data, and those that did not report the adjusted effect sizes. We used the Newcastle Ottawa Scale for quality assessment. Random-effect model meta-analysis was performed. Publication bias was assessed by Begg’s test.

**Results:**

Overall, nine studies comprising a total of 61,104 participants were included. There was a significant positive association between metabolic syndrome and MI among those with obesity (hazard ratio (HR): 1.68; 95% confidence interval (CI): 1.27, 2.22). Subgroup analysis showed higher HRs for obesity (1.72; 1.03, 2.88) than overweight (1.58; 1.-13-2.21). Meta-regression revealed no significant association between nationality and risk of MI (*p* = 0.75). All studies had high qualities. There was no significant publication bias (*p* = 0.42).

**Conclusions:**

Metabolic syndrome increased the risk of MI in those with EBW. Further studies are recommended to investigate other risk factors of CVDs in EBW, in order to implement preventive programs to reduce the burden of CVD in obesity.

**Supplementary Information:**

The online version contains supplementary material available at 10.1186/s12889-024-17707-7.

## Introduction

Cardiovascular diseases (CVDs) are a major cause of morbidity and mortality in developed and developing countries and are accounting for 46.2% of total deaths worldwide [1]. As a risk factor for CVDs, metabolic syndrome is a disorder defined by the co-occurrence of at least three of five medical conditions, which are hyperglycemia, elevated triglyceride (TG), hypertension, low high-density lipoprotein (HDL), and obesity [2]. Along with lifestyle changes, metabolic syndrome is becoming a more serious health issue as the number of obese patients constantly increases among children and adults [3, 4]. Metabolic syndrome is associated with several debilitating outcomes, such as myocardial infarction (MI), diabetes, and stroke [5]. Additionally, metabolically healthy obese individuals are at a higher risk of MI than metabolically healthy individuals with normal weight [6].

Several prior studies have been conducted to identify the association between metabolic syndrome and MI, all of which have shown that metabolic syndrome is an important risk factor for MI [1, 7, 8]. It is believed that lifestyles and nutritional factors, especially excess body weight (EBW) and insufficient physical activity play important roles in hypertension, hyperglycemia, dyslipidemia, and ultimately, MI development [1, 9].

However, there are controversial findings in the studies regarding the association between metabolic syndrome and CVDs. Moreover, studies were conducted on different populations and in different settings [1, 10, 11]. Although several studies suggested a positive association between metabolic syndrome and MI in individuals with obesity [12, 13], others reported contradictory results [14, 15]. So, opinions regarding the impact of metabolic syndrome on MI in people with EBW or metabolically unhealthy obese patients are debatable. It is important to note that while meta-analyses are carried out to examine the association between metabolic syndrome and CVDs [16, 17], none have examined the association between metabolically unhealthy obesity and MI, nor have they been published in recent years. Therefore, there is a need to conduct a pooled analysis to make a conclusive statement about the association between metabolic syndrome and CVDs in those with EBW. This systematic review and meta-analysis aimed to investigate both whether there is an association between metabolic syndrome and MI in individuals with EBW and to investigate the strength of the association using meta-analysis while reporting the pooled effect size of the association.

## Methods

The study was conducted according to the guidelines of the Preferred Reporting Items for Systematic reviews and Meta-Analyses 2020 [18].

### Study design and eligibility criteria

We included data from studies evaluated the association between metabolic syndrome and MI among participants with overweight or obesity, collectively mentioned as EBW. The PICO framework was as follow: Population: Individuals with EBW; Intervention/exposure: Diagnosis of metabolic syndrome using valid criteria; Comparison: Individuals with normal body mass index (BMI); and Outcomes: MI.

Cohort studies that evaluated the association between metabolic syndrome and MI in individuals with EBW without applying any limitation on age, sex, language, and ethnicity were included. Studies with incomplete or unavailable original data, reanalysis of previously published data, and those that did not report the adjusted effect size of the association between metabolic syndrome and outcomes of interest were excluded. Moreover, clinical trials, case reports, editorials, reviews, news, book chapters, and retracted articles were excluded. In the cases where outcomes were published at different time points, the last evaluation was considered.

### Database searching and study selection

We searched electronic databases, including PubMed/Medline, Scopus, and Web of Science. Initially, keywords were selected using medical subject headings and screening of related articles and journals. Then, searches were performed separately in the databases from January 1, 2010 to June 30, 2021. We also updated the search on December 9, 2023. The detailed search quaery for each database is presented in Table S1.

The search records were imported into the Mendeley software and deduplicated using that software. Then, two independent reviewers screened the titles and abstract. In the next step, the full-texts of the articles were retrieved and evaluated by the same reviewers. Discrepancies were resolved by consultation with the principal investigator. If the data could not be extracted from the study, we emailed the corresponding authors three times with a one week interval and asked to provide the data. If we did not receive a response or they did not provide such results, we excluded those studies.

### Data extraction and risk of bias assessment

Data were extracted and summarized in a predefined data extraction form in Microsoft Excel software. In case of disagreement between the two reviewers, the third reviewer was consulted. The extracted data included study characteristics (i.e., first author’s name, publication year, follow-up, country, and study type), population characteristics (i.e., sample size, sex, age, systolic and diastolic blood pressures, fasting blood sugar (FBS), TG, HDL, low-density lipoprotein (LDL), waist circumferences, BMI, and history of smoking) and outcomes. If a study reported the results as a graph, data were extracted by “data extraction from graph method” explained by Sistrom and Mergo [19].

The risk of bias assessment was performed using the nine-star Newcastle Ottawa scale (NOS), including selection (representativeness of the population), comparability of groups (adjustment for confounders such as age and sex), and ascertainment of outcomes [20]. The NOS assigns four stars for selection, two for comparability, and three for outcome. The NOS scores of more than seven were acknowledged as high quality [20].

### Statistical analysis

The STATA version 14.0 (Stata Corporation, College Station, TX) was used for statistical analysis. We used the “metan” command to perform a pooled analysis (a random or fixed effect analysis based on the heterogeneity among studies). Findings were presented as an overall hazard ratio (HR) with a 95% confidence interval (95% CI). Heterogeneity among studies was assessed using the Q-statistic and I-square test, and *p*-values less than 0.05 or I-square > 50% were considered as high heterogeneity. In case of high heterogeneity, subgroup analysis and meta-regression were used to investigate the potential source of heterogeneity. Funnel plot was only used to evaluate publication bias if at least ten studies were included [21]. Also, Begg's test was used to identify publication bias [22].

## Results

**Fig. 1:**
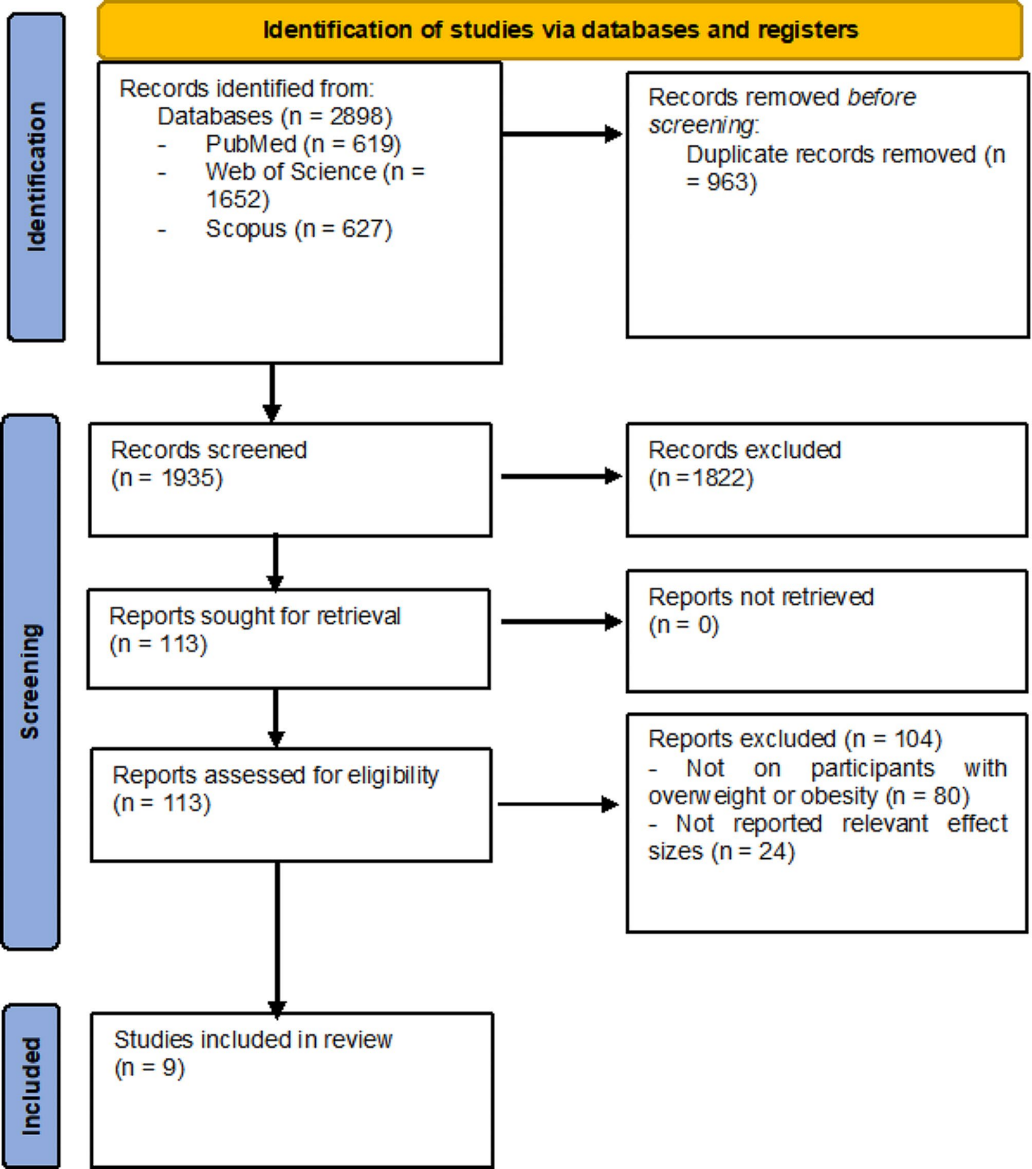
Study selection process

The search found 2898 results. Following removing 963 duplicates, 1935 articles were included for the title/abstract screening. Then, 113 studies were included for the full-text reviewing. Finally, the data from nine studies were included in the meta-analysis [6, 23–30]. Eighty studies were excluded because they were not conducted on individuals with EBW and 24 studies were excluded because the adjusted effect sizes were not reported (Fig. 1).

### Study characteristics

These studies included 61104 participants from eight different countries and regions. The follow-up duration ranged from one to 11.6 years. The studies were published between 2010 and 2023. Three studies used adult treatment panel III (ATP-III) [25–27], while others used other definitions like Japanese society of internal medicine [6], American heart association/National heart, lung, and blood institute [24], harmonized international diabetes federation (IDF) [23], World Health Organization [28], joint interim statement [29], and IDF [30] (Table 1).

**Table 1:** Characteristic of the included studies

First author	Year	Country/continent	Follow up duration (year)	Sample size	Definition of metabolic syndrome
Wataru Hirokawa	2010	Japan	11.0	242	JSIM
Leon Simons	2011	Australia	1.0	194	AHA/NHLBI
Alexandra Ogorodnikova	2012	United States	11.6	1167	ATP-III
Mette Thomsen	2013	Denmark	8.0	7080	ATP-III
Seung Hun Lee	2018	Korea	10.0	2460	ATP-III
Yijie Xu	2018	China	1.0	1167	HIDF
Laura Sánchez-Iñigo	2016	Europe	10.0	426	WHO
Jiacheng Ding	2023	China	1.9	46,055	JIS
Jacob Opio	2022	Australia	9.7	2313	IDF

Abbreviations: JSIM: Japanese Society of Internal Medicine; AHA/NHLBI: American Heart Association/National Heart, Lung and Blood Institute; ATP-III: Adult Treatment Panel III; HIDF: Harmonized International Diabetes Federation; WHO: World Health Organization^;^ JIS: Joint Interim Statement; IDF: International Diabetes Federation

The study by Ogorodnikova et al. [25] was conducted on obese participants (BMI: 33.7 kg/m2) compared to the study by Lee et al. which was conducted on people with overweight [27]. The average of TG was lower in the study by Ogorodnikova et al. than Lee et al. (96.0 mg/dl vs. 189.9 mg/dl). Systolic blood pressures (SBPs) were 122.5, 131.8, and 150.0 mmHg in the studies by Ogorodnikova et al., Lee et al., and Thomsen et al., respectively [25–27]. FBS was 95.3 mg/dl in the Ogorodnikova’s study [25] compared to 179.2 mg/dl in Lee’s study [27] and 97.0 mg/dl in Thomsen’s study [26]. In addition, HDL values were 56.8, 39.6, and 46.0 mg/dl in Ogorodnikova et al., Lee et al., and Thomsen et al., respectively [25–27] (Table 2).

**Table 2:** Baseline characteristics of the participants in Lee, Thomsen, and Ogorodnikova studies

Variables	Seung Hun Lee	Mette Thomsen	Alexandra Ogorodnikova
Mean age (year)	56.2	58.0	55.5
Sex (Male %)	100%	55%	32.2%
BMI	27.4	33.00	33.7
SBP	131.8 ± 28.8	150 (137–162)	122.5 ± 18.2
DBP	81.5 ± 17.9	89 (82–96)	74.4 ± 10.6
HDL	39.6 ± 15.9	46(39–54)	56.8 ± 13.5
LDL	119.3 ± 38.8	131(108–158)	134.7 ± 36.4
TG	189.9 ± 144.9	212(177–283)	96.00 ± 44
FBS	179.2 ± 73.4	97.0(88.0-108.0)	95.3 ± 8.4
Diabetes	26.9%	11.0%	3.3%

Abbreviations: BMI: Body mass index; SBP: Systolic blood pressure; DBP: Diastolic blood pressure; HDL: High density lipoprotein; LDL: Low density lipoprotein; TG: Triglyceride; FBS: Fasting blood sugar

### Quality assessment and publication bias

All studies had a high quality. The quality assessment scores were seven in three studies, eight in five studies, and nine in one study [27]. All studies had a high quality regarding selection of non-exposed cohorts, ascertainment of exposure, controlling for confounders, and duration of follow-up. The risk of bias assessment showed that seven studies did not report data regarding report the adequacy of a follow-up cohort (Table 3).

**Table 3:** Quality assessment results based on Newcastle-Ottawa scale for cohorts

		Selection				Comparability		Outcome			
Author	Year	Representativeness of the exposed cohort	Selection of the non-exposed cohort	Ascertainment of exposure	Demonstration that outcome of interest was not present at start of study	The study controls for age, sex and marital status	Study controls for other factors	Assessment of outcome	Was follow-up long enough for outcomes to occur	Adequacy of follow-up of cohorts	Total
Seung Hun Lee et al.	2018	*	*	*	*	*	*	*	*	*	9
Yijie Xu et al.	2018	*	*	*	*	*	*	*	*	No statement	8
Laura Sa´nchez-In˜igo, et al.	2016	*	*	*	*	*	*	No statement	*	No statement	7
Mette Thomsen et al.	2013	*	*	*	*	*	*	*	*	No statement	8
Alexandra et al.	2012	*	*	*	*	*	*	*	*	No statement	8
Leon A et al.	2011	*	*	*	-	*	*	*	*	No statement	7
Wataru Hirokawa et al.	2010	-	*	*	*	*	*	*	*	No statement	7
Jiacheng Ding et al.	2023	*	*	*	*	*	*	*	*	No statement	8
Jacob Opio et al.	2022	*	*	*	*	*	*	*	*	-	8

The Begg's test showed no significant publication bias (*p* = 0.42).

### Overall meta-analysis results

**Fig. 2:**
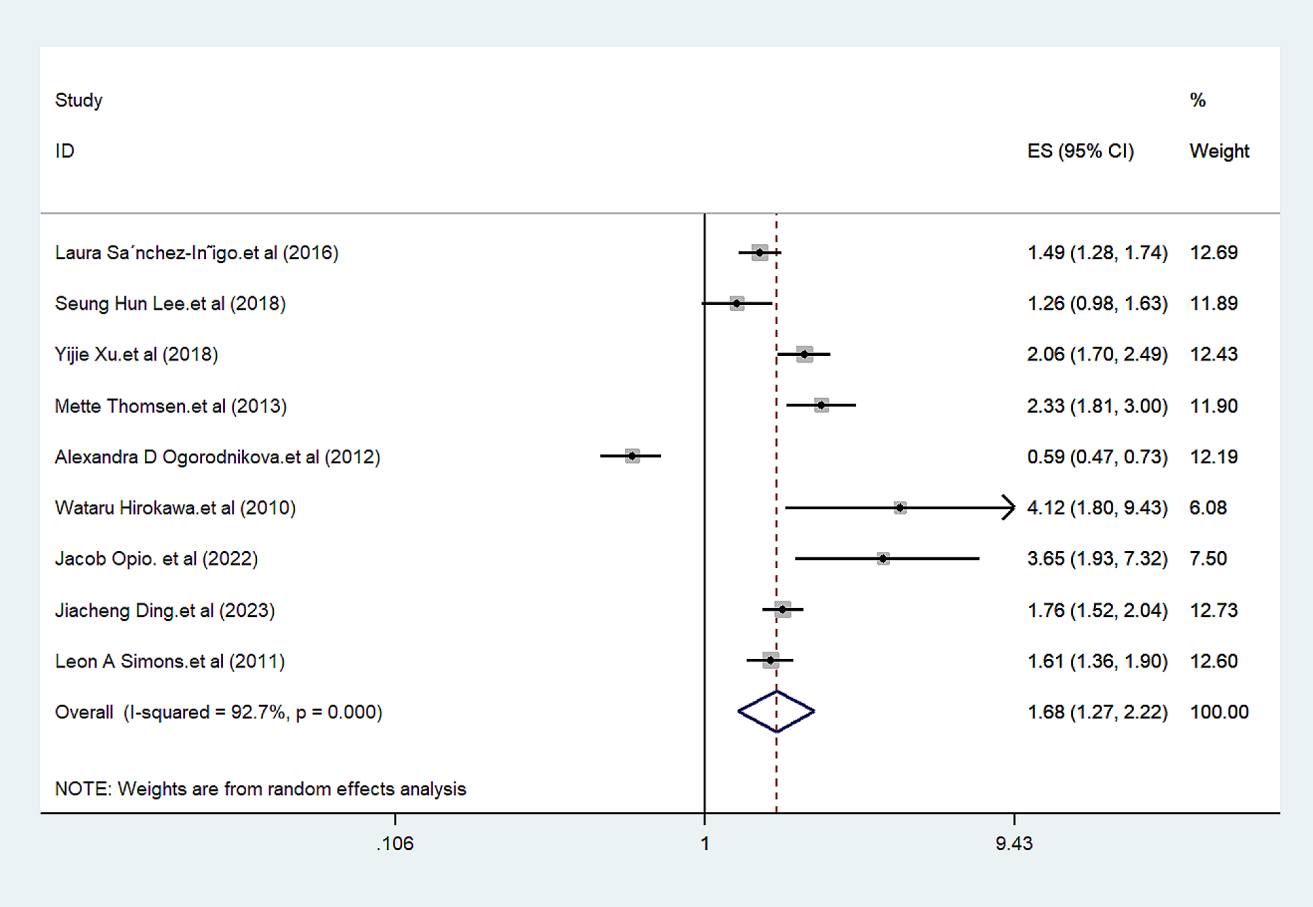
Forest plots of the association between metabolic syndrome and myocardial infarction among individuals with excess body weight. ES: effect size; CI: confidence interval

We found a significant positive association between metabolic syndrome and MI among obese patients (HR = 1.68; 95% CI: 1.27, 2.22). Among nine studies included in the analysis, only one study showed a significant negative association between metabolic syndrome and MI (HR = 0.59; 95% CI: 0.47, 0.73) (Fig. 2).

### Subgroup analysis and meta-regression

**Fig. 3:**
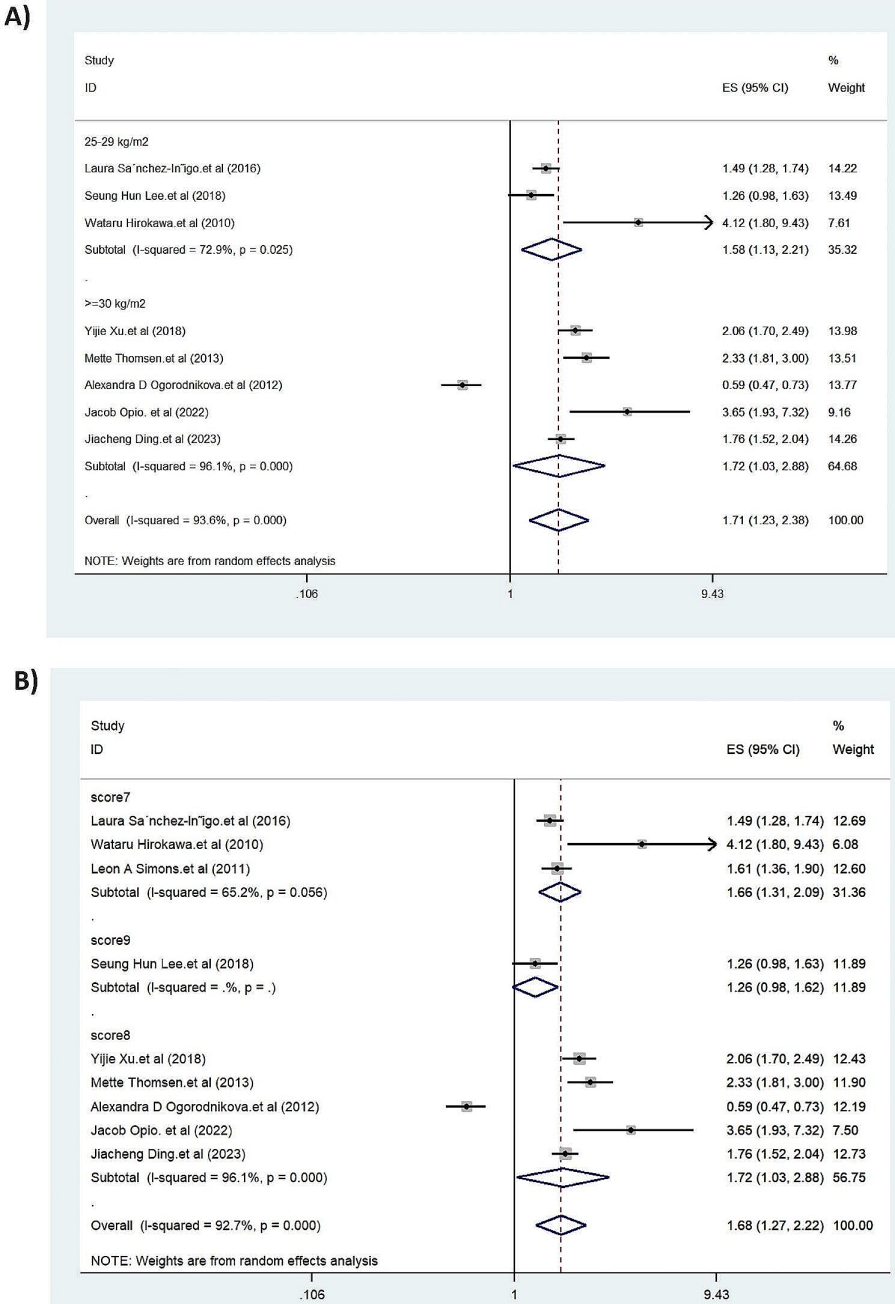
Forest plots of the association between metabolic syndrome and myocardial infarction among individuals with excess body weight by body mass index values (**A**) and quality assessment scores (**B**). ES: effect size; CI: confidence interval

We performed subgroup analysis by quality assessment scores and BMI values. The pooled HRs for overweight (25 < BMI ≤ 29.9 kg/m2) and obesity (BMI ≥ 30 kg/m2) were 1.58 (95% CI: 1.13, 2.21) and 1.72 (95% CI: 1.03, 2.88), respectively (Fig. 3A). Subgroup analysis by quality assessment scores showed higher pooled HRs for score eight (1.72; 95% CI: 1.03, 2.88) than score seven (1.66; 95% CI: 1.31, 2.09) (Fig. 3B). The meta-regression showed no significant association between nationality and risk of MI (*p* = 0.75).

## Discussion

To the best of our knowledge, no previous meta-analyses have assessed the association between metabolic syndrome and MI among individuals with EBW. Our results suggested that metabolic syndrome increased the risk of MI by 1.68 times among patients with EBW. The effect size was higher for obesity compared with overweight.

Among the nine studies included, only one study reported a negative association between metabolic syndrome and MI in patients with EBW [25]. In this regard, the article by Lavie and colleagues proposed a debate that some studies showed a better prognosis for CVDs in people with EBW than those with normal weights [31]. Nevertheless, the overall findings of our meta-analysis showed a significant higher risk of MI in people with EBW and metabolic syndrome. Also, previous studies showed adverse effects of metabolic syndrome. Accordingly, metabolic syndrome increased the risk of major adverse cardiovascular events by 1.55 times (95% CI: 1.28, 1.87) in patients with hypertension [32]. Another meta-analysis on eight studies showed that patients with end-stage renal disease and metabolic syndrome had an increased risk of mortality (risk ratio (RR): 1.92; 95% CI: 1.15, 3.21) and CVDs (RR: 6.42; 95% CI: 2.00, 20.58) compared to those without metabolic syndrome [33]. Therefore, it appears that metabolic syndrome has remarkable negative effects on risk of MI. Nevertheless, other large scale studies on people with EBW are recommended.

We found a high heterogeneity between studies (I-square: 92.7%). To account for the source of heterogeneity, we performed meta-regression and subgroup analysis. Meta-regressions showed no significant association with nationality. Also, subgroup analysis by quality assessment and BMI determined no source for heterogeneity. So, this heterogeneity might be related to the received treatments and relevant drugs that were not specifically reported in the primary studies. In this regard, the paper by Ogorodnikova et al. mentioned that the components of metabolic syndrome were controlled through medications [25].

It is worth noticing that people who are involved in the cohort studies might be different from healthy people in the general population because those who participated in the cohort study are under both drug and non-drug treatment, especially in obese patients. In addition, people with obesity are more taken under control, and their disease is under treatment. Due to this fact, metabolic syndrome is a protective factor for CVDs in this study. Interestingly, among different factors, the country is considered an important special contributor to that protective association. It is noticeable that the pattern of obesity is different among different countries [34]. For example, the average BMI in the United States is higher than other countries [35]. In that regard, patients with metabolic syndrome who reside in China, Japan, and Korea may not need any treatment although they have symptoms of metabolic syndrome. As a result, the severity of metabolic syndrome varies from one country to another [36]. Considering all these explanations, they did not require any drug treatments due to the early diagnosis of participants’ metabolic syndrome at the primary stages. On the other hand, the severity of metabolic syndrome in the United States was high, and all patients underwent drug treatments. Accordingly, this might explain the reasons for the protective results found in the study by Ogorodnikova et al., which was conducted in the United States [25].

### Strengths and limitations

The strength of the study lies in that it is one of the pioneer studies that was focused on people with EBW and evaluated the association between metabolic syndrome and MI among them. We used a robust meta-analytical approach to report the pooled effect size for this association. Also, our included cohort studies were of high quality.

 Additionally, the issue of confounders was controlled by including only cohort studies and using adjusted HRs in the analysis. So, the findings can be valuable for health policymaking and clinicians for prevention and reduction the mortality and morbidity of CVDs, particularly MI, in individuals with EBW.

Nevertheless, this systematic review and meta-analysis has some limitations that need to be taken into consideration when interpreting the results. First, the number of studies included in this meta–analysis was low. Therefore, we could not assess the publication bias using a funnel plot. Moreover, there was a high heterogeneity. To find the potential sources of heterogeneity, we performed subgroup analysis and meta-regression. However, due to the small sample number of included studies, the heterogeneities remained high. Second, a large proportion of studies did not provide sufficient information about the effect sizes among participants, leading to their exclusion. Third, although the included studies performed adjusted analysis based on several factors, there is still a possibility of biases due to inadequate adjustment for confounders. Fourth, in most primary studies, medical records were used for data gathering, raising the possibility of misclassification. Although we searched three major online databases, we did not perform grey literature search, thus potentially missing unpublished data.

## Conclusions

Overall, metabolic syndrome significantly increased the risk of MI by 68% among individuals with EBW. Therefore, the findings of the study can be used by health policymakers to develop preventive programs for patients with EBW. Also, physicians should control the relevant risk factors, especially metabolic syndrome, in order to prevent from MI in individuals with EBW. Further large-scale observational studies and meta-analyses are needed to determine other risk factors of CVDs in patients with EBW, especially in other countries and populations like African countries and the African American race.

### Electronic supplementary material

Below is the link to the electronic supplementary material.

Supplementary Material 1

## Data Availability

The datasets generated and/or analyzed during the current study are not publicly available due to decision of the research team but are available from the corresponding author on reasonable request.

## Abbreviations

CVD: Cardiovascular disease
HDL: High-density lipoprotein
MI: Myocardial infarction
EBW: Excess body weight
BMI: Body mass index
FBS: Fasting blood sugar
TG: Triglyceride
LDL: Low-density lipoprotein
NOS: Newcastle Ottawa scale
HR: Hazard ratio
CI: Confidence interval
ATP-III: Adult treatment panel III
IDF: International diabetes federation
RR: Risk ratio
