# Supplementary material for: Association between metabolic syndrome and myocardial infarction among patients with excess body weight: a systematic review and meta-analysis
Source: BMC Public Health. 2024 Feb 12;24:444. doi: 10.1186/s12889-024-17707-7 (PMC10863149; doi:10.1186/s12889-024-17707-7)
Supplement: Supplementary file 1 — Supplementary Material 1 [file 12889_2024_17707_MOESM1_ESM.doc]

**Table S1.** Search strategy for PubMed, Scopus, and Web of Science.

| Database | Query | Results (Search date: December 9, 2023) |
| --- | --- | --- |
| PubMed | (“Metabolic Syndrome”[tiab] OR (Syndrome[tiab] AND Metabolic[tiab]) OR (Syndromes[tiab] AND Metabolic[tiab]) OR “Metabolic Syndrome X”[tiab] OR (Syndrome X[tiab] AND Metabolic[tiab]) OR “Metabolic X Syndrome”[tiab] OR (Syndrome[tiab] AND Metabolic X[tiab]) OR (X Syndrome[tiab] AND Metabolic[tiab]) OR “Dysmetabolic Syndrome X”[tiab] OR (Syndrome X[tiab] AND Dysmetabolic[tiab]) OR “Metabolic Cardiovascular Syndrome”[tiab] OR (Cardiovascular Syndrome[tiab] AND Metabolic[tiab]) OR (Syndrome[tiab] AND Metabolic Cardiovascular[tiab]) OR “Cardiometabolic Syndrome”[tiab] OR (Syndrome[tiab] AND Cardiometabolic[tiab])) AND ((“Transluminal Coronary”[tiab] AND “Balloon Dilation”[tiab]) OR (“Balloon Dilation”[tiab] AND “Coronary Artery”[tiab]) OR PCI[tiab] OR (Angioplasty[tiab] AND “Coronary Balloon”[tiab]) OR (Angioplasties[tiab] AND “Coronary Balloon”[tiab]) OR (“Balloon Angioplasties”[tiab] AND Coronary[tiab]) OR (“Balloon Angioplasty”[tiab] AND Coronary[tiab]) OR “Coronary Balloon Angioplasties”[tiab] OR “Coronary Balloon Angioplasty”[tiab] OR (“Coronary Angioplasty”[tiab] AND “Transluminal Balloon”[tiab]) OR (Angioplasty[tiab] AND Transluminal[tiab] AND “Percutaneous Coronary”[tiab]) OR “Percutaneous Transluminal Coronary Angioplasty”[tiab] OR (“Artery Bypass”[tiab] AND Coronary[tiab]) OR (“Artery Bypasses”[tiab] AND Coronary[tiab]) OR (Bypasses[tiab] AND “Coronary Artery”[tiab]) OR “Coronary Artery Bypasses”[tiab] OR “Coronary Artery Bypass Surgery”[tiab] OR (Bypass[tiab] AND “Coronary Artery”[tiab]) OR “Aortocoronary Bypass”[tiab] OR “Aortocoronary Bypasses”[tiab] OR CABG[tiab] OR (Bypass[tiab] AND Aortocoronary[tiab]) OR (Bypasses[tiab] AND Aortocoronary[tiab]) OR (“Bypass Surgery”[tiab] AND “Coronary Artery”[tiab]) OR “Coronary Artery Bypass Grafting”[tiab] OR “Myocardial Infarction” [tiab] OR “Cardiovascular Stroke”[tiab] OR (Stroke[tiab] AND Cardiovascular[tiab]) OR “Myocardial Infarct”[tiab] OR (Strokes[tiab] AND Cardiovascular[tiab]) OR (Infarct[tiab] AND Myocardial[tiab]) OR “Myocardial Infarcts”[tiab] OR “Heart Attack”[tiab]) AND (“Abdominal Obesity”[tiab] OR (Obesity[tiab] AND Abdominal[tiab]) OR “Central Obesity”[tiab] OR (Obesity[tiab] AND Central[tiab]) OR (Obesity[tiab] AND Visceral[tiab]) OR “Visceral Obesity”[tiab] OR obes*[tiab] OR overweight[tiab] OR (“Benign Obesity”[tiab] AND Metabolically[tiab]) OR “Metabolically Healthy Obesity”[tiab] OR (“Healthy Obesity”[tiab] AND Metabolically[tiab]) OR (Obesity[tiab] AND “Metabolically Healthy”[tiab]) OR “Metabolically Benign Obesity”[tiab]) AND 2010/01/01:2023/12/09[dp] | 619 |
| Scopus | (TITLE-ABS(“Metabolic Syndrome”) OR (TITLE-ABS(Syndrome) AND TITLE-ABS(Metabolic)) OR (TITLE-ABS(Syndromes) AND TITLE-ABS(Metabolic)) OR TITLE-ABS(“Metabolic Syndrome X”) OR (TITLE-ABS(Syndrome X) AND TITLE-ABS(Metabolic)) OR TITLE-ABS(“Metabolic X Syndrome”) OR (TITLE-ABS(Syndrome) AND TITLE-ABS(Metabolic X)) OR (TITLE-ABS(X Syndrome) AND TITLE-ABS(Metabolic)) OR TITLE-ABS(“Dysmetabolic Syndrome X”) OR (TITLE-ABS(Syndrome X) AND TITLE-ABS(Dysmetabolic)) OR TITLE-ABS(“Metabolic Cardiovascular Syndrome”) OR (TITLE-ABS(Cardiovascular Syndrome) AND TITLE-ABS(Metabolic)) OR (TITLE-ABS(Syndrome) AND TITLE-ABS(Metabolic Cardiovascular)) OR TITLE-ABS(“Cardiometabolic Syndrome”) OR (TITLE-ABS(Syndrome) AND TITLE-ABS(Cardiometabolic))) AND ((TITLE-ABS( “Transluminal Coronary”) AND TITLE-ABS(“Balloon Dilation”)) OR (TITLE-ABS(“Balloon Dilation”) AND TITLE-ABS(“Coronary Artery”)) OR TITLE-ABS(PCI) OR (TITLE-ABS(Angioplasty) AND TITLE-ABS(“Coronary Balloon”)) OR (TITLE-ABS(Angioplasties) AND TITLE-ABS(“Coronary Balloon”)) OR (TITLE-ABS(“Balloon Angioplasties”) AND TITLE-ABS(Coronary)) OR (TITLE-ABS(“Balloon Angioplasty”) AND TITLE-ABS(Coronary)) OR TITLE-ABS(“Coronary Balloon Angioplasties”) OR TITLE-ABS(“Coronary Balloon Angioplasty”) OR (TITLE-ABS(“Coronary Angioplasty”) AND TITLE-ABS(“Transluminal Balloon”)) OR (TITLE-ABS(Angioplasty) AND TITLE-ABS(Transluminal) AND TITLE-ABS(“Percutaneous Coronary”)) OR TITLE-ABS(“Percutaneous Transluminal Coronary Angioplasty”) OR (TITLE-ABS(“Artery Bypass”) AND TITLE-ABS(Coronary)) OR (TITLE-ABS(“Artery Bypasses”) AND TITLE-ABS(Coronary)) OR (TITLE-ABS(Bypasses) AND TITLE-ABS(“Coronary Artery”)) OR TITLE-ABS(“Coronary Artery Bypasses”) OR TITLE-ABS(“Coronary Artery Bypass Surgery”) OR (TITLE-ABS(Bypass) AND TITLE-ABS(“Coronary Artery”)) OR TITLE-ABS(“Aortocoronary Bypass”) OR TITLE-ABS(“Aortocoronary Bypasses”) OR TITLE-ABS(CABG) OR (TITLE-ABS(Bypass) AND TITLE-ABS(Aortocoronary)) OR (TITLE-ABS(Bypasses) AND TITLE-ABS(Aortocoronary)) OR (TITLE-ABS(“Bypass Surgery”) AND TITLE-ABS(“Coronary Artery”)) OR TITLE-ABS(“Coronary Artery Bypass Grafting”) OR TITLE-ABS(“Myocardial Infarction”) OR TITLE-ABS(“Cardiovascular Stroke”) OR (TITLE-ABS(Stroke) AND TITLE-ABS(Cardiovascular)) OR TITLE-ABS(“Myocardial Infarct”) OR (TITLE-ABS(Strokes) AND TITLE-ABS(Cardiovascular)) OR (TITLE-ABS(Infarct) AND TITLE-ABS(Myocardial)) OR TITLE-ABS(“Myocardial Infarcts”) OR TITLE-ABS(“Heart Attack”)) AND (TITLE-ABS(“Abdominal Obesity”) OR (TITLE-ABS(Obesity) AND TITLE-ABS(Abdominal)) OR TITLE-ABS(“Central Obesity”) OR (TITLE-ABS(Obesity) AND TITLE-ABS(Central)) OR (TITLE-ABS(Obesity) AND TITLE-ABS(Visceral)) OR TITLE-ABS(“Visceral Obesity”) OR TITLE-ABS(obes*) OR TITLE-ABS(overweight) OR (TITLE-ABS(“Benign Obesity”) AND TITLE-ABS(Metabolically)) OR TITLE-ABS(“Metabolically Healthy Obesity”) OR (TITLE-ABS(“Healthy Obesity”) AND TITLE-ABS(Metabolically)) OR (TITLE-ABS(Obesity) AND TITLE-ABS(“Metabolically Healthy”)) OR TITLE-ABS(“Metabolically Benign Obesity”)) AND (PUBYEAR > 2010 AND PUBYEAR < 2024) | 627 |
| Web of sciences | (TS=(“Metabolic Syndrome”) OR (TS=(Syndrome) AND TS=(Metabolic)) OR (TS=(Syndromes) AND TS=(Metabolic)) OR TS=(“Metabolic Syndrome X”) OR (TS=(Syndrome X) AND TS=(Metabolic)) OR TS=(“Metabolic X Syndrome”) OR (TS=(Syndrome) AND TS=(Metabolic X)) OR (TS=(X Syndrome) AND TS=(Metabolic)) OR TS=(“Dysmetabolic Syndrome X”) OR (TS=(Syndrome X) AND TS=(Dysmetabolic)) OR TS=(“Metabolic Cardiovascular Syndrome”) OR (TS=(Cardiovascular Syndrome) AND TS=(Metabolic)) OR (TS=(Syndrome) AND TS=(Metabolic Cardiovascular)) OR TS=(“Cardiometabolic Syndrome”) OR (TS=(Syndrome) AND TS=(Cardiometabolic))) AND ((TS=(“Transluminal Coronary”) AND TS=(“Balloon Dilation”)) OR (TS=(“Balloon Dilation”) AND TS=(“Coronary Artery”)) OR TS=(PCI) OR (TS=(Angioplasty) AND TS=(“Coronary Balloon”)) OR (TS=(Angioplasties) AND TS=(“Coronary Balloon”)) OR (TS=(“Balloon Angioplasties”) AND TS=(Coronary)) OR (TS=(“Balloon Angioplasty”) AND TS=(Coronary)) OR TS=(“Coronary Balloon Angioplasties”) OR TS=(“Coronary Balloon Angioplasty”) OR (TS=(“Coronary Angioplasty”) AND TS=(“Transluminal Balloon”)) OR (TS=(Angioplasty) AND TS= (Transluminal) AND TS=(“Percutaneous Coronary”)) OR TS=(“Percutaneous Transluminal Coronary Angioplasty”) OR (TS=(“Artery Bypass”) AND TS=(Coronary)) OR (TS=(“Artery Bypasses”) AND TS=(Coronary)) OR (TS=(Bypasses) AND TS=(“Coronary Artery”)) OR TS=(“Coronary Artery Bypasses”) OR TS=(“Coronary Artery Bypass Surgery”) OR (TS=(Bypass) AND TS=(“Coronary Artery”)) OR TS=(“Aortocoronary Bypass”) OR TS=(“Aortocoronary Bypasses”) OR TS=(CABG) OR (TS=(Bypass) AND TS=(Aortocoronary)) OR (TS=(Bypasses) AND TS=(Aortocoronary)) OR (TS=(“Bypass Surgery”) AND TS=(“Coronary Artery”)) OR TS=(“Coronary Artery Bypass Grafting”) OR TS=(“Myocardial Infarction”) OR TS=(“Cardiovascular Stroke”) OR (TS=(Stroke) AND TS=(Cardiovascular)) OR TS=(“Myocardial Infarct”) OR (TS=(Strokes) AND TS=(Cardiovascular)) OR (TS=(Infarct) AND TS=(Myocardial)) OR TS=(“Myocardial Infarcts”) OR TS=(“Heart Attack”)) AND (TS=(“Abdominal Obesity”) OR (TS=(Obesity) AND TS=(Abdominal)) OR TS=(“Central Obesity”) OR (TS=(Obesity) AND TS=(Central)) OR (TS=(Obesity) AND TS=(Visceral)) OR TS=(“Visceral Obesity”) OR TS=(obes*) OR TS=(overweight) OR (TS=(“Benign Obesity”) AND TS=(Metabolically)) OR TS=(“Metabolically Healthy Obesity”) OR (TS=(“Healthy Obesity”) AND TS=(Metabolically)) OR (TS=(Obesity) AND TS=(“Metabolically Healthy”)) OR TS=(“Metabolically Benign Obesity”)) AND PY=(2010-2021) | 1652 |
